# Rapid methods to create a positive control and identify the *PAX8/PPARγ* rearrangement in FNA thyroid samples by molecular biology

**DOI:** 10.18632/oncotarget.24995

**Published:** 2018-04-10

**Authors:** Emilia Vuttariello, Elio Biffali, Raimondo Pannone, Anna Capiluongo, Mario Monaco, Valentina Sica, Concetta Aiello, Marco Matuozzo, Maria Grazia Chiofalo, Gerardo Botti, Gennaro Chiappetta

**Affiliations:** ^1^ Functional Genomics Unit, Istituto Nazionale Tumori -IRCCS -Fondazione G. Pascale, Napoli, Italia; ^2^ Molecular Biology and Bioinformatics Unit, Stazione Zoologica “A. Dorhn”, Naples, Italy; ^3^ CMO, Naples, Italy; ^4^ Thyroid Surgery Unit, Istituto Nazionale Tumori -IRCCS -Fondazione G. Pascale, Napoli, Italia; ^5^ S.S.D. di Citopatologia e S.C. di Anatomia Patologica, Istituto Nazionale Tumori -IRCCS -Fondazione G. Pascale, Napoli, Italia

**Keywords:** thyroid, FNA (fine needle aspiration), PAX8 gene, PPAR gene, PAX8/PPARγ rearrangement

## Abstract

Thyroid cancer is the most common malignancy of the endocrine system and includes well-differentiated forms, namely papillary and follicular carcinomas, and the poorly differentiated and undifferentiated forms that result from the transformation of thyroid follicular cells (anaplastic carcinomas). Notably, 5–10% of all thyroid cancers are medullary thyroid cancers that arise from parafollicular cells also known as C cells. The most common genetic mutations in papillary and follicular thyroid cancers are point mutations of the *BRAF* or *RAS* genes, while the most common chromosomal alterations are *RET/PTC* and *PAX8/PPARγ* rearrangements. The most frequent initial manifestation of thyroid cancer is the appearance of a nodule most of which are benign; indeed, less than 5% are malignant. However, some cases are misdiagnosed, and many patients undergo unnecessary surgery. Therefore, an accurate pre-surgery evaluation is crucial. The most reliable diagnostic test for thyroid nodules is fine needle aspiration (FNA) cytology, which accurately distinguishes between a benign and malignant lesion in most cases. However, cytological discrimination between malignant and benign follicular cancer is often difficult because of poor quality samples. Here we describe rapid methods to create a positive control and identify the *PAX8/PPARγ* rearrangement in FNA thyroid samples by molecular biology.

## INTRODUCTION

Thyroid cancer is the most common malignant tumor of the endocrine system [1]. Most cancers derive from thyroid follicular cells and include well-differentiated forms, namely, papillary and follicular carcinomas and poorly differentiated forms such as anaplastic carcinoma [2]. About 15% of all thyroid cancers are follicular thyroid cancers (FTC) [3, 4]. Follicular carcinoma is considered more aggressive than papillary carcinoma. It occurs only rarely after radiation exposure, and generally in a slightly older age group than papillary thyroid cancer and is also less common in children.

The most frequent initial manifestation of thyroid cancer is the appearance of a nodule (more than 20% of the population has a palpable thyroid nodule and the percentage increases to 70% with ultrasound). In the great majority of cases (95%) it is simply an hyperplasia or a benign lesion, and only 5–30% of nodules are malignant and require surgical treatment. The most reliable diagnostic test for thyroid nodules is fine needle aspiration (FNA). Although this procedure clearly differentiates benign lesions from malignant lesions in about 60–70% of cases, cytology is “uncertain” in 20–30% of cases [5]. In these cases, a second FNA procedure or even surgery may be necessary, which results in additional morbidity and higher healthcare costs. Moreover, patients with malignant and indeterminate FNA cytology typically undergo limited surgery (i.e. lobectomy). If malignancy is diagnosed by pathological examination of the excised nodule, patients must undergo a second operation to complete the thyroidectomy, which, again, is associated with additional morbidity and costs. In addition, 1–3% of nodules diagnosed as benign on FNA are later found to be malignant on follow-up (false-negative FNA), and the delay in treatment before a definitive diagnosis results in high of risk of disease progression.

Molecular biology techniques are increasingly being used in routine cancer diagnostics [6–8]. Four molecular alterations, *BRAF* and *RAS* point mutations, and *RET/PTC* and *PAX8/PPAR*γ rearrangements, are the most frequent causes of thyroid papillary and follicular carcinomas [9–11]. The most frequent genetic alterations in follicular carcinomas are point mutations of *RAS* and the *PAX8/PPARγ* rearrangement. These two alterations have been identified in approximately 80% of cases, and they seem to be mutually exclusive, which supports the hypothesis that they are distinct molecular subtypes. Moreover they are found in 18–52% of follicular carcinomas and in 24–53% of follicular adenomas. A much lower incidence has been reported in Hürthle cell tumors (15–25% of carcinomas and 0–4% of adenomas) [12].

The *PAX8/PPARγ* fusion oncogene [13] has been identified only in follicular carcinomas and in a small fraction (about 13%) of follicular adenomas [14]. Paired box 8 (*PAX8*), localized on chromosome 2q13, encodes the thyroid-specific paired domain transcription factor that is essential for the differentiation of follicular cells and the regulation of thyroid-specific genes. *PAX8* is formed by 12 exons. Alternative splicing of exons 8–10 results in the production of multiple protein isoforms. The peroxisome proliferator–activated receptor gamma (*PPARγ*) gene, localized on chromosome 3p25 belongs to the nuclear receptor family of transcription factors that regulates cell differentiation and lipid metabolism. The two genes can fuse via translocation (2q:3p)(13:25) to form a new fusion gene that expresses a PAX8/PPARγ fusion protein, designated “PPFP”. Typically, the translocation fuses *PAX8* intron 10 with the intron immediately preceding the first coding exon of *PPARγ*. Alternative splicing produces multiple RNA isoforms within the same neoplasia.

It is difficult, being a enough rare rearrangement, to have a *PAX8/PPARγ* fusion positive sample to use as positive control and for this reason we have made a sample suitable for this purpose.

Here we describe a rapid method to create a positive control and identify the rearrangement in one experiment in FNA thyroid samples by PCR.

## RESULTS AND DISCUSSION

**Figure 1:**
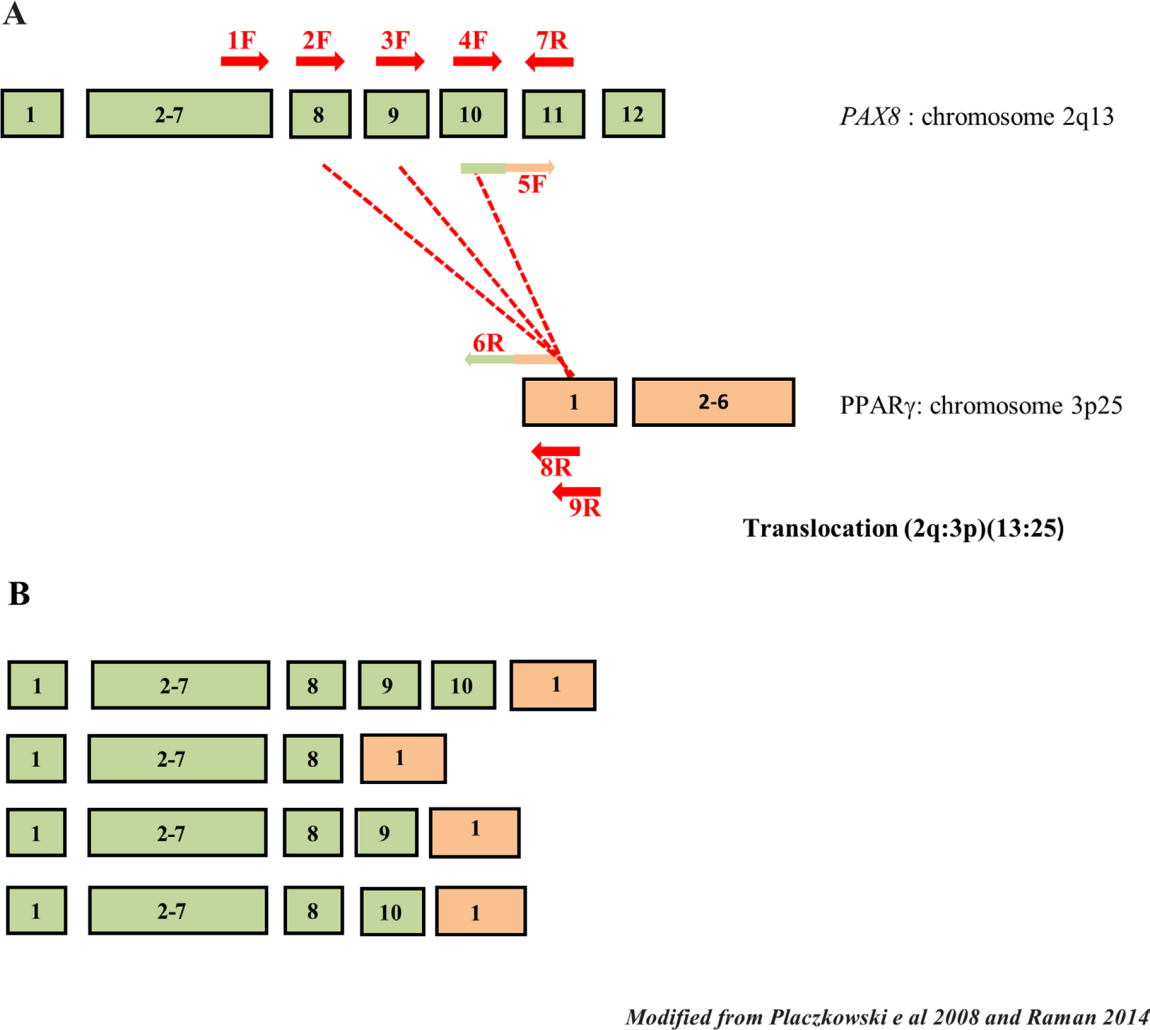
Schematic drawing of *PAX8* splicing and *PAX8/PPARγ* rearrangements (**A**) The *PAX8* and *PPARγ* exons involved in the *PAX8/PPARγ* rearrangements. Exons 8, 9 and 10 of *PAX8* are affected by alternative splicing. The primers used in our methodologies are reported in red (in different colors only the primers with the tail). (**B**) In rearrangement *PAX8* alternative splicing can result in multiple forms in the same tumor. It is shown the more common rearrangements maintaining the reading frame.

The *PAX8* gene is formed by 12 exons but the first exon isn’t translated, alternative splicing of exon 8–10 results in different form that can be present simultaneously in the same tissue. The *PAX8/PPARγ* rearrangement is generally due to the translocation between *PAX8* intron 10 and intron *PPARγ* immediately preceding exon 1. In Figure 1 we show a plain picture of the *PAX8/PPARγ* gene rearrangement and alternative splicing of *PAX8*. In Figure 1A are positioned, marked in red, the oligonucleotides we used, while in Figure 1B we presented the multiple rearrangement *PAX8/PPARγ* forms produced by alternative splicing all maintaining the open reading frame. Specifically, *PAX8* exons 1–8 (exons 9 and 10 deleted), 1–9 (deleted exon 10 deleted) and 1–10 (exon 9 deleted) have been detected fused to the first coding exon of *PPARγ*, and all these situations maintain the *PPARγ* open reading frame [15].

**Figure 2:**
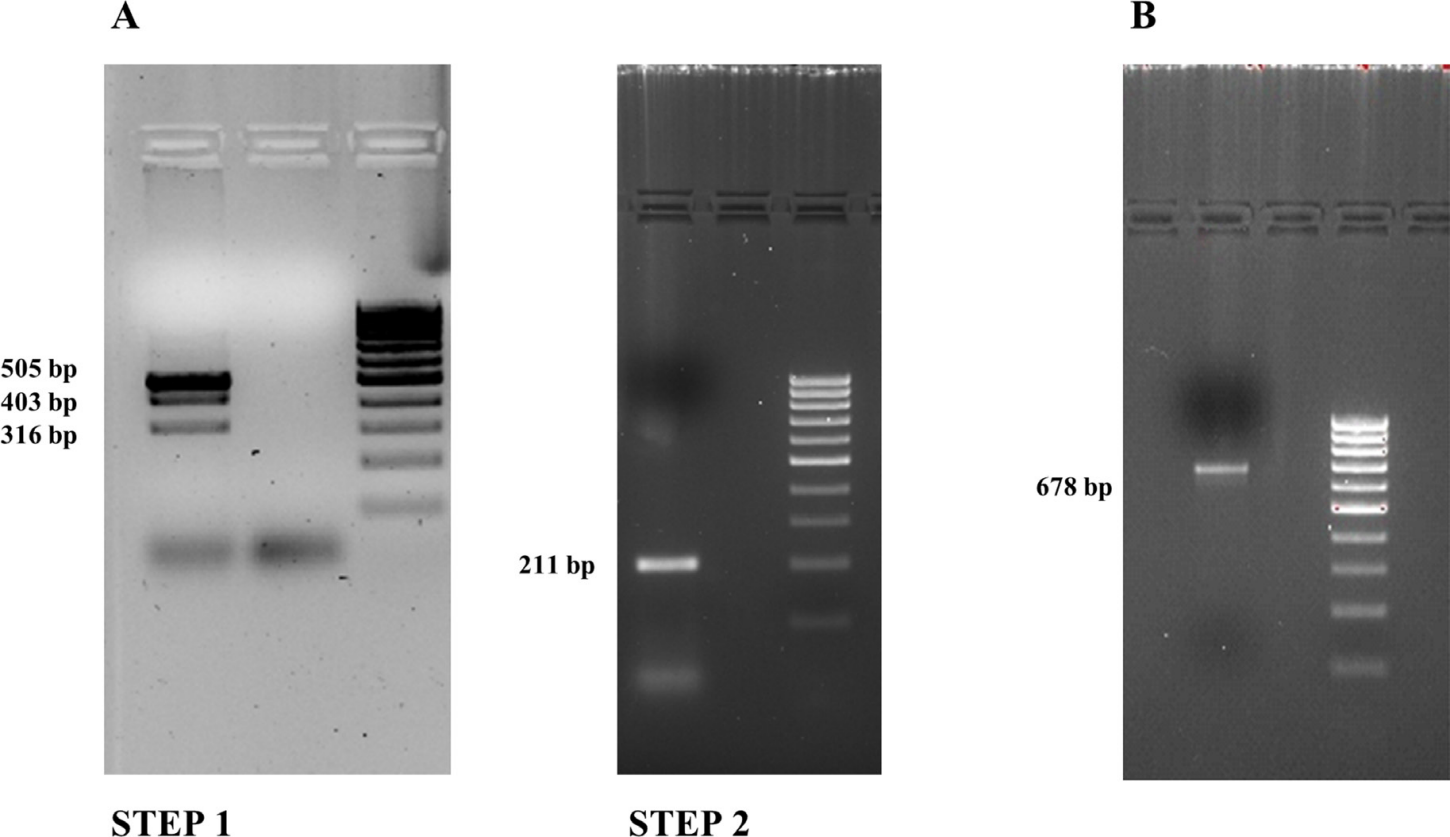
Overlapping PCR results (2% agarose gel) (**A**) Step 1: *PAX8* amplification bands: 505 bp, 403 bp (no exon 10) and 316 bp (no exon 9) fragments. Step 2: *PPARγ* amplification band: 211 bp. (**B**) The *PAX8/PPARγ* fusion gene.

Because the rarity of the *PAX8/PPARγ* rearrangement and the lack of cell lines carrying this mutation, we had to create a positive control to identify this chromosomic alteration. We approached this problem starting from wild-type thyroid RNA using the overlapping PCR technique, which is a simple, versatile technique for site-directed mutagenesis and gene splicing. We used two primers to join two DNA molecules: for each molecule, one primer is constructed such that it has a 5’ overhang complementary to the end of the other molecule. After the annealing, when the replication occurs, the DNA is extended to a new sequence that is complementary to the molecule to which it will be joined. Once both DNA molecules are extended, they are mixed and a PCR is carried out with only the primers for the far ends. The initial PCRs generate fragments with tails that are used as a template DNA to create a full-length product. The two stages of our overlapping PCR procedure are shown in Figure 2. As shown in Figure 2A we carried out two different amplifications: in step 1 the amplification of the *PAX8* gene resulted in a 505 bp band, and in bands measuring 403 bp (no exon 10) and 316 bp (no exon 9) attributable to alternative splicing, whereas amplification of the *PPARγ* gene resulted in a 211 bp fragment (step 2). Then we pooled and amplified 10 μl of the products of steps 1 and 2 obtaining 5 fragments measuring 678 bp, 576 bp, 489 bp, 316 bp and 211bp, respectively (data no shown). In Figure 2B we show the longest purified amplification fragment (678 bp) that we used as positive control because it covers the whole rearrangement region. The fragment sizes from rearrangements are approximate because these processes can occur in different exon positions.

**Figure 3:**
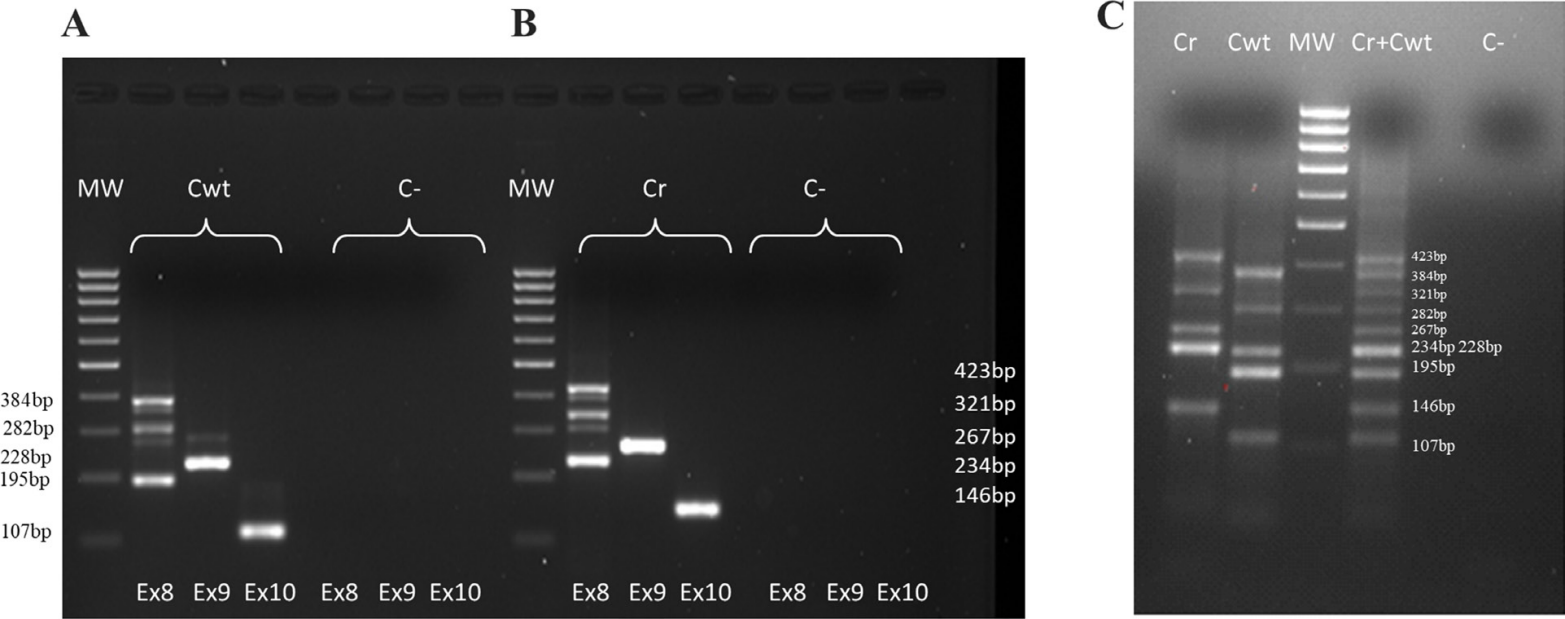
Positive controls amplifications (**A**) Wild type *PAX8* positive control (Cwt): *PAX8* forms amplifications (forward primers respectively on *PAX8* exon 8, exon 9 and exon 10 and reverse primer on *PAX8* exon 11). (**B**) *PAX8/PPARγ* rearrangement positive control (Cr): *PAX8/PPARγ* rearrangement forms amplifications (forward primers respectively on *PAX8* exon 8, exon 9 and exon 10 and reverse primer on *PPARγ* exon 1). (**C**) Cwt, Cr and (Cwt + Cr) amplifications in multiplex (simultaneously forward primers on *PAX8* exon 8, exon 9 and exon 10 and reverse primers on *PAX8* exon 11 and *PPARγ* exon 1)

To set-up our multiplex method we have performed some steps: in the first step we have amplified Cwt in three different reactions using forward primers positioned respectively on exons 8, 9 and 10 of the *PAX8* gene and reverse primer on *PAX8* exon 11 and, as shown in Figure 3A, the amplification with the forward primer on *PAX8* exon 8 resulted in multiple products (384 bp, 282 bp and 195 bp) originating, as mentioned above, from alternative splicing, while a 228 bp band appeared when we use a forward primer on *PAX8* exon 9 and a 107 bp band appeared when we used an oligonucleotide as forward primer on *PAX8* exon 10. Then we have amplified Cr using forward primers respectively on *PAX8* exon 8, exon 9 and exon 10 and reverse primer on *PPARγ* exon 1. In Figure 3B are shown the amplification results: with the forward primer on *PAX8* exon 8 we got multiple products (423 bp, 321 bp and 234 bp), with the forward primer on *PAX8* exon 9 we obtained a 267 bp band and with the forward primer on *PAX8* exon 10 a 146 bp band. Lastly we carried out a multiplex amplification in which we mixed three forward primers and two different reverse primers. The results obtained on positive controls are reported in Figure 3C.

**Figure 4:**
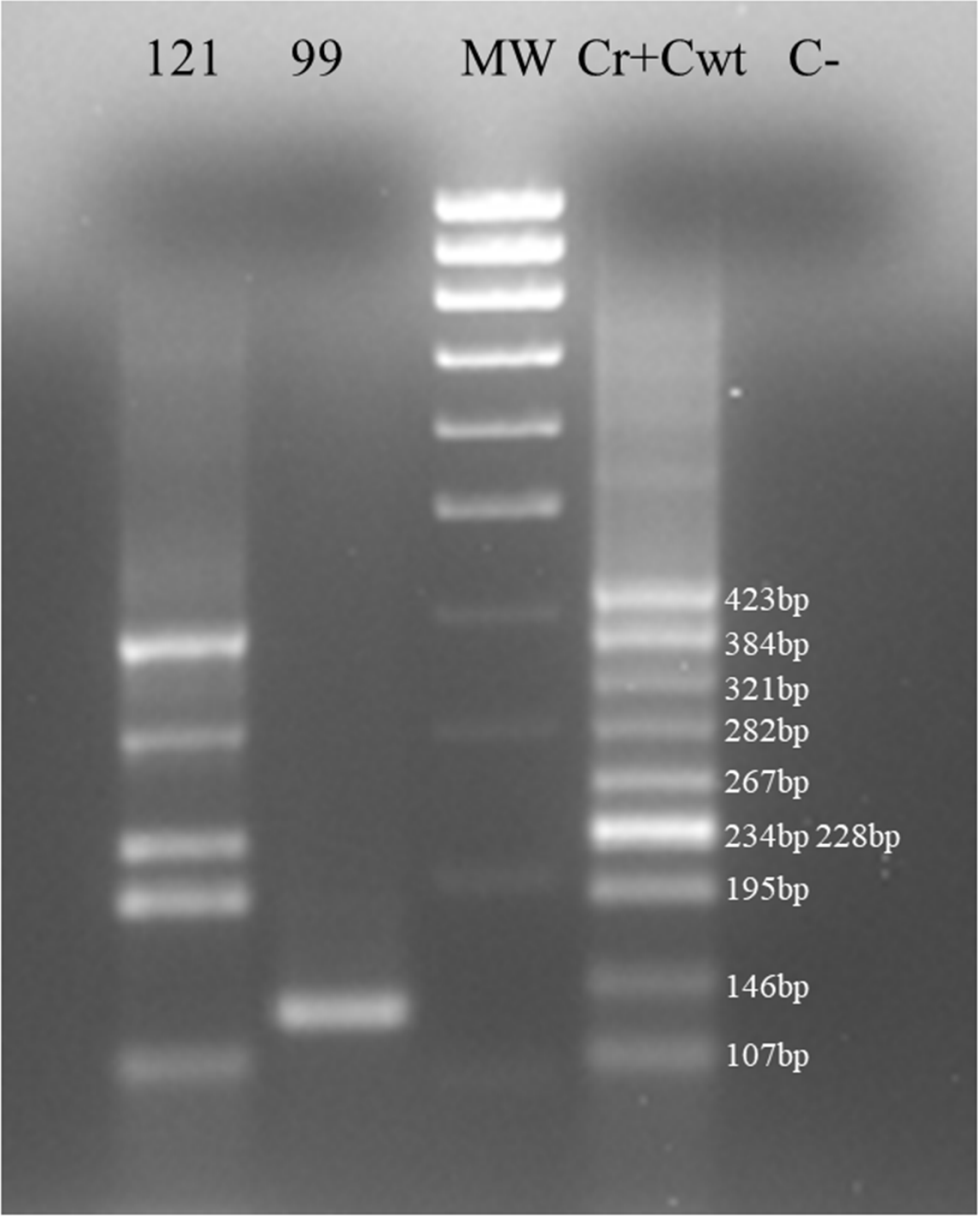
Multiplex samples amplification Multiplex amplifications of sample 121, sample 99 and (Cwt + Cr). Sample 99 was negative for *PAX8* wild-type amplification and positive for *PAX8/PPARγ* rearrangement.

In Figure 4 we show the last procedure with patients samples and we study with a single experiment both wild type *PAX8* and for *PAX8/PPARγ* rearrangement. In fact it is possible to distinguish in the figure that the sample 121 is wild-type *PAX8* positive and the sample 99 is affected by the *PAX8/PPARγ* rearrangement.

**Figure 5:**
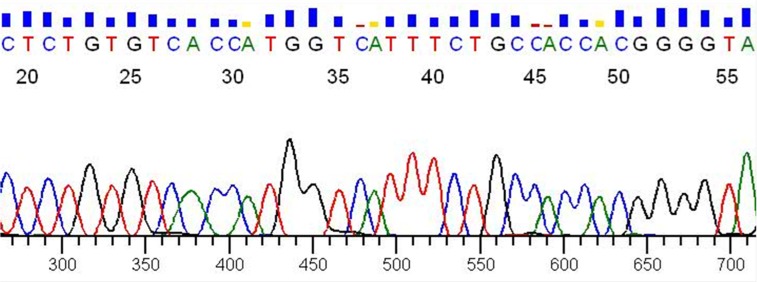
Sanger sequencing Verification by Sanger sequencing of the reverse sequence of sample 99 shown in Figure 4.

This result was confirmed by Sanger sequencing (Figure 5, reverse sequencing). The fusion sample shows a translocation between *PAX8* exon 8 and *PPARγ* exon 1 with a deletion of *PAX8* exons 9 and 10 by alternative splicing and for this reason we did not obtain any result for sequences with the forward primer on exon 9 or on exon 10. Using this strategy we analyzed 60 RNA from FNA thyroid samples but only 4 were positive for the chromosomal rearrangement.

The *PAX8/PPARγ* fusion protein has been implicated in thyroid follicular oncogenesis because it abrogates normal PPARγ function [16], increases cell cycle transition, reduces apoptosis and induces loss of both anchorage-dependence and contact inhibition [17].

The protein product of *PAX8*/*PPARγ* acts, at least in part, by inhibiting wild-type PPARγ signaling, which suggests that FTCs that present this rearrangement could be sensitive to PPARγ agonist therapy. Chromosomal alterations of *PPARγ* that result in the expression of the fusion protein PPFP may be an early event in the development or progression of FTC, and perhaps, the *PAX8/PPARγ* rearrangement could not be sufficient for the development of a full malignant phenotype. Indeed, additional genetic or epigenetic events may be required to enable the full phenotypic expression of FTCs [18].

Here we describe rapid inexpensive methods to create a positive control and to identify the wild type *PAX8* and *PAX8/PPARγ* rearrangements in one experiment in FNA thyroid samples by PCR. Both methods are feasible even in laboratories that do not have sophisticated equipment or highly experienced staff. The described procedures could help to determine diagnosis. In addition, the identification of *PAX8* gene alteration could help to develop selective and personalized therapy.

## MATERIALS AND METHODS

### Routine sample collection

We performed routine ultrasound-guided FNA using a 23 gauge needle. The aspirated sample was used for cytology and the residual material and the needle wash were directly collected into a tube containing 1 ml of RLT buffer (Qiagen, Germantown, MD, USA) and 10 µL 2-mercaptoethanol. The tube was stored frozen at –80° C until extraction of nucleic acid. Nucleic acid extraction was performed according to the manufacturer’s instruction (Qiagen RNeasy mini kit). Quantity and quality were assessed with a NanoDrop 1000 spectrophotometer (Thermo Scientific, Waltham, MA, USA), 1 μg of RNA was reverse transcribed into cDNA using the “QuantiTect Reverse Transcription kit” (Qiagen). The cDNA was stored at –20° C. GAPDH a housekeeping gene was used to verify that all the RNA samples were suitable for molecular analysis.

### *PAX8/PPARγ* fusion positive control

**Figure 6:**
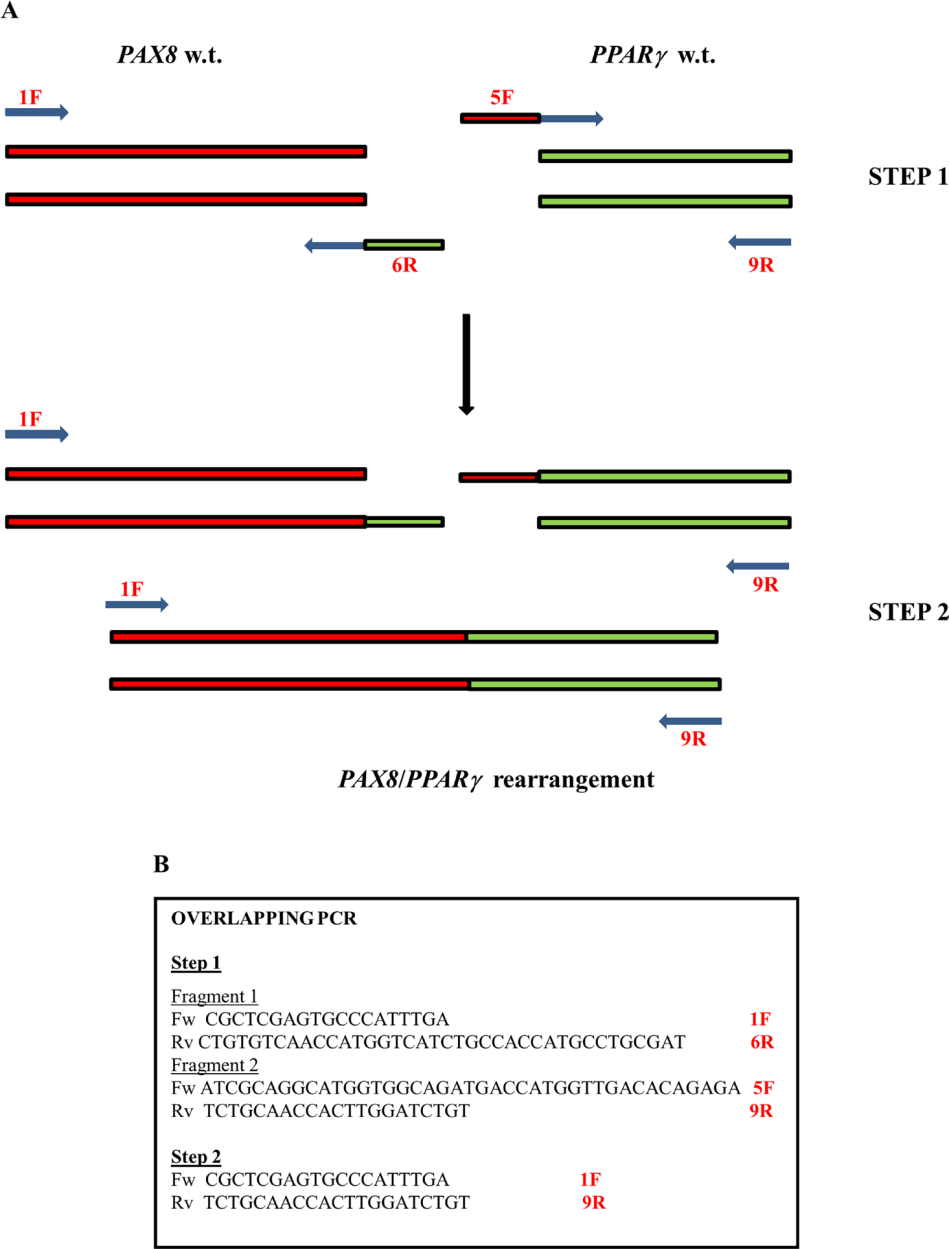
The plan of overlapping PCR (**A**) The two-step overlapping PCR procedure used to obtain a *PAX8/PPARγ* fusion positive control. (**B**) The panel shows the primers used.

To obtain a *PAX8/PPARγ* fusion positive control, we carried out a two-step overlapping PCR procedure (Figure 6A). In the first step we amplified the *PAX8* and *PPARγ* genes. The primers for *PAX8* amplification were located on exons 7 and 10 indicated respectively 1F and 6R in Figures 1 and 6. The primer on exon 10 was built with a tail corresponding to a region of exon 1 of *PPARγ,* whereas the primers for *PPAR*γ amplification were located on *PPARγ* exon 1 but the forward primer was linked to a region of *PAX8* exon 10 (5F and 9R Figures 1 and 6). Thus, the amplification products each bear nucleotide tails that correspond to the other studied gene. In Figure 6B we list overlapping PCR used primers. We performed PCR in a 25 μl final volume using as template 250 ng of cDNA product obtained by RT-PCR starting from 1 μg of total RNA. The amplification mixture consisted of 1× PCR buffer (Roche, Pleasanton, CA, USA), 200 μmol of dNTP and 1U of Taq DNA polymerase (Roche) and 0.2 pmol/μl of forward and reverse primers. The PCR thermal profile consisted of initial denaturation at 94° C for 10 min, followed by 40 cycles: denaturation at 95° C for 30 s, annealing at 48° C for 30 s and extension at 72° C for 1 min. Final extension was at 72° C for 10 min.

In the second step we used the same *PAX8* forward primer and *PPARγ* reverse primer used in the first amplification. We also used the same mixture as in the first step but added, as template, 10 μl of product obtained from *PAX8* amplification and 10 μl of product obtained from *PPARγ* amplification. The PCR thermal profile was designed to foster the formation of a hybrid *PAX8* and *PPARγ* product. Initial denaturation, at 94° C for 10 min, was followed by 10 cycles consisting of denaturation at 95° C for 30 s, annealing at 48° C for 30 s and extension at 72° C for 1 min, and then by 30 cycles consisting of denaturation at 95° C for 30 s, annealing at 52° C for 30 s and extension at 72° C for 1 min. Final extension was at 72° C for 10 min. The amplification products were controlled on a 2% agarose gel. We next cloned the fusion gene using the Stratagene StrataClone PCR cloning kit (La Jolla, CA, USA), and extracted the plasmid DNA using the GenElute HP plasmid miniprep kit by Sigma-Aldrich (St Louis, MO, USA) according to the supplier’s instructions.

### Wilde type *PAX8* and *PAX8/PPARγ* fusion gene analysis

**Figure 7:**
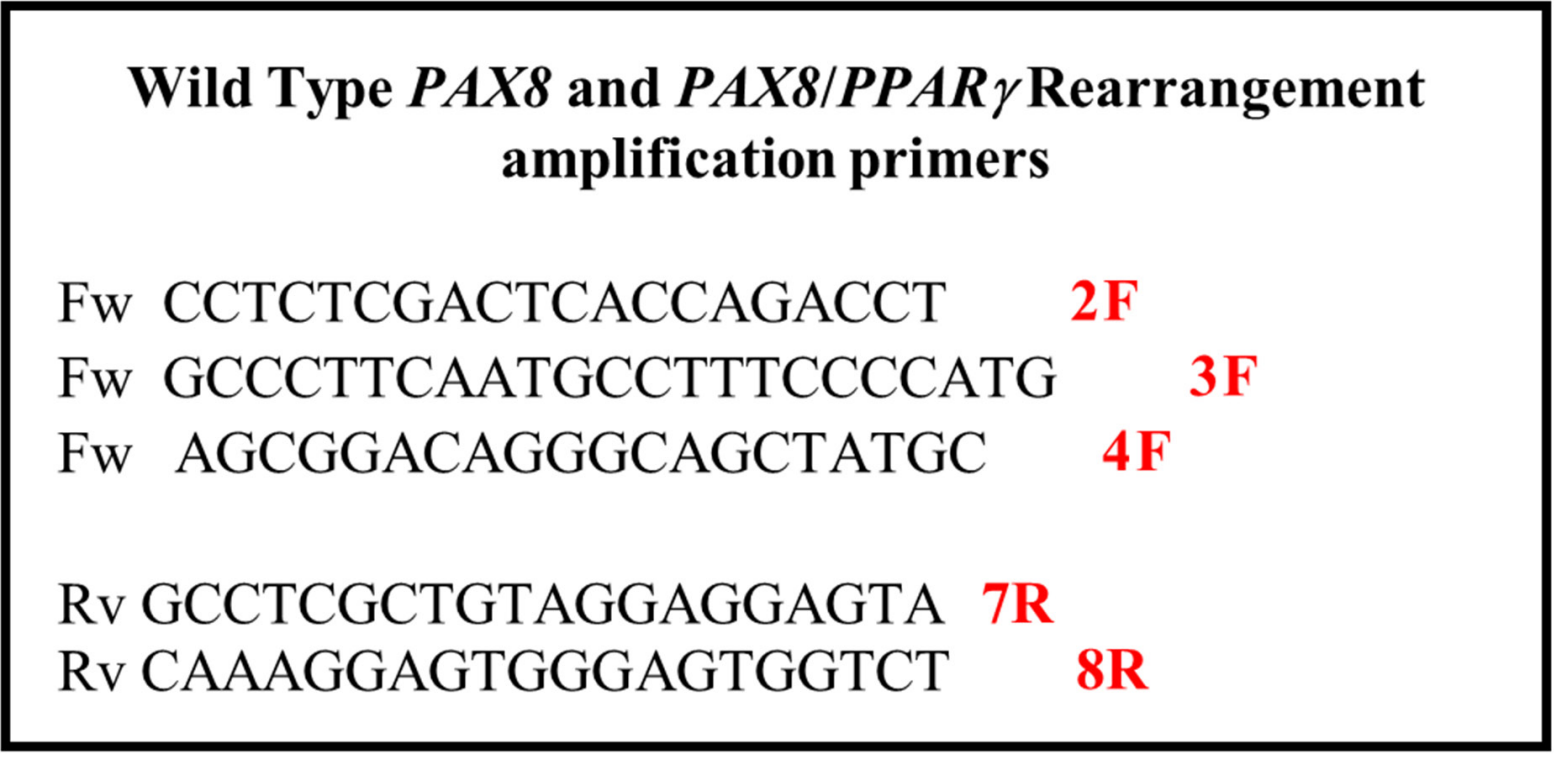
Primer’s list Primers used for multiplex amplification of wild type *PAX8* and *PAX8/PPARγ* rearrangement.

Samples, previously extracted and evaluated with the ubiquitous GAPDH gene, and controls, one positive wild type PAX8 sample (Cwt) and for the *PAX8/PPARγ* rearrangement, the fragment obtained by the overlapping PCR (Cr), are amplified for the wild-type *PAX8* gene using the forward primers positioned respectively on exons 8, exon 9, exon 10 and reverse primer on exon 11 of the *PAX8* gene, while for the *PAX8/PPARγ* amplification the forward primer is the same of previous reaction but the reverse primer is on exon 1 of *PPARγ.* The PCR conditions are the same described in overlapping PCR procedure while the PCR thermal profile consisted of initial denaturation at 94° C for 10 min, followed by 30 cycles: denaturation at 95° C for 30 s, annealing at 60° C for 30 s and extension at 72° C for 1 min. Final extension was at 72° C for 10 min. All PCR amplifications are performed with the C-1000 Thermal Cycler (Bio-Rad, CA, USA). In the same conditions we set up the simultaneous amplification of the three different *PAX8* isoform both in Cwt than in Cr adding in mix forward primers on *PAX8* exon 8, exon 9 and exon 10. Then we make a multiplex amplification of wild type *PAX8* and *PAX8/PPARγ* rearrangement. The amplification conditions are the same as previously described but the annealing time is 1 min 30 s and reverse primers concentration, exon 11 of the *PAX8* gene and on exon 1 of *PPARγ*, is modified to 0.3 pmol/μl. The amplifications are gel controlled (2% agarose gel in 1X TBE) and rearrangement-positive PCR products are purified (High Pure PCR Product Purification Kit Roche) and sequenced on both strands (forward and reverse) according to the manual of the Applied Biosystem (Waltham, MA, USA) “Big dye Terminator v3.1 Cycle Sequencing kit”. All sequencing amplification reactions of the individual *PAX8/PPARγ* rearrangement regions were performed using forward primers positioned respectively on exons 8, 9 and 10 of the *PAX8* gene, while the reverse primer is the same for all the three amplifications positioned on exon 1 of *PPARγ.* The primers used, for both amplification and sequencing reactions, modified from Algeciras-Schimnich *et al.* [19], are listed in Figure 7. Subsequently, the sequence reactions were purified by Agencourt CleanSeq Beckman Coulter system (Brea, CA, USA) and analyzed by capillary electrophoresis on the Applied Biosystems “3730 DNA Analyzer”.
